# Plasma Menthol Glucuronide as a Biomarker for the Behavioral Effects of Menthol and Nicotine in Humans

**DOI:** 10.3389/fphar.2022.844824

**Published:** 2022-03-31

**Authors:** Ralitza Gueorguieva, Elizabeth K. C. Schwartz, R. Ross MacLean, Elise E. DeVito, Tore Eid, Ran Wu, Stephanie S. O’Malley, Mehmet Sofuoglu

**Affiliations:** ^1^ Yale School of Public Health, Department of Biostatistics, New Haven, CT, United States; ^2^ Yale University, School of Medicine, Department of Psychiatry, New Haven, CT, United States; ^3^ VA Connecticut Healthcare System, West Haven, CT, United States; ^4^ Yale University, School of Medicine, Department of Laboratory Medicine, New Haven, CT, United States

**Keywords:** nicotine, addiction, menthol, menthol glucuronide, plasma menthol

## Abstract

This secondary analysis sought to determine if plasma menthol glucuronide (MG) concentrations predict changes in three outcomes, subjective drug effects, urges to smoke, and heart rate, following concurrent inhaled menthol and intravenous nicotine. A total of 45 menthol and non-menthol cigarettes smokers (36 male, nine female, 20 Black, and 23 White) were included in this double-blind, placebo-controlled study. Across three test sessions, participants were assigned to a different flavor condition for each session: 0% (no menthol), 0.5%, or 3.2% menthol. In each test session, participants received in a random order one intravenous delivery of saline and two intravenous deliveries of nicotine (0.25 mg/70 kg and 0.5 mg/70 kg), each 1 h apart, concurrent with menthol delivery by e-cigarettes. The main outcomes were subjective drug effects, urges to smoke, and heart rate. The results showed that following e-cigarette inhalation, changes in plasma MG concentrations or “menthol boost” increased proportionally to the menthol concentration in the e-liquids. While changes in plasma MG concentrations were not predictive of increases in heart rate or subjective drug effects that are reflective of acute effects from nicotine (i.e., *feel good effects, stimulated, aversive effects*), they were predictive of *cooling effect*, a typical effect of menthol, but only in menthol smokers in the absence of concurrent active nicotine infusion. These findings demonstrate the utility of plasma MG as a biomarker both for acute menthol exposure by e-cigarette inhalation and for the examination of the concentration-dependent behavioral and physiological effects of menthol in humans.

## Introduction

Nicotine is a relatively weak primary reinforcer when compared to other drugs of abuse. Consequently, the initiation and maintenance of nicotine addiction may require exposure to additional reinforcers that are added to tobacco products (Caggiula et al., 2008). Menthol, a commonly used flavoring agent in various tobacco products, may serve this role (Hans et al., 2012; Biswas et al., 2016). Menthol’s cooling and anti-irritant effects in the airways may counteract the aversive effects of nicotine and tobacco smoke, especially among youth, who are experimenting with cigarettes (Hersey et al., 2006; Wise et al., 2012). Preference for menthol cigarettes continues to increase, especially among minority and vulnerable populations, while the overall rate of cigarette smoking has decreased in the United States (Villanti et al., 2016; Cwalina et al., 2020). Therefore, the impact of menthol flavoring on the initiation and maintenance of cigarette smoking remains a public health concern (Dennis and Scott, 2007; Mills et al., 2020).

The anti-irritant and cooling effects of menthol are through activation of the transient receptor potential melastatin 8 (TRPM8) receptors (Peier et al., 2002), which are expressed in the mucosa lining of the upper and lower airways, mouth, nose, and throat. Menthol may also affect nicotine reinforcement through nicotinic acetylcholine receptors (nAChR); however, systemic effects of menthol have not been well characterized (Wickham, 2020). Following its intake, menthol is rapidly metabolized to menthol glucuronide (MG), which has been shown to be a biomarker for mentholated cigarette smoking status (Gelal et al., 1999; Benowitz et al., 2010). Similarly, increases in plasma MG concentrations have been documented following menthol cigarette smoking, also called the “menthol boost” (Hsu et al., 2017). We have also reported that the area under the curve (AUC) values of plasma MG concentrations following e-cigarette use are proportional to the menthol concentrations in the e-liquid (Jatlow et al., 2018). To our knowledge, previous studies have not examined whether plasma MG levels serve as a biomarker for the acute subjective and physiological effects of menthol-flavored tobacco products.

In this study, we present a secondary analysis of plasma MG concentrations that were measured in a prior analysis examining acute responses to concurrently administered menthol and nicotine in menthol and non-menthol smokers (Valentine et al., 2018). In three test sessions, menthol was administered via e-cigarettes at three different concentrations: 0% (no menthol), 0.5% menthol, or 3.2% menthol. Concurrent with each e-cigarette inhalation, one of three different nicotine doses was infused intravenously: saline, 0.25 mg/70 kg nicotine and 0.5 mg/70 kg nicotine. In each test session, participants received their assigned menthol concentration combined with each of the three nicotine doses in a random order. The rationale for combining IV nicotine with inhaled menthol was to minimize the conditioned responses to inhaled nicotine combined with menthol, as observed in cigarette or e-cigarette use (Valentine et al., 2018). IV nicotine infusion allowed assessment of acute, dose-dependent subjective and physiological nicotine effects in smokers independent from chemosensory cues (e.g., mouth, throat, and upper airways) associated with nicotine inhalation. By using an e-cigarette to deliver menthol without nicotine, the local sensory cues produced by menthol were maintained, without the chemosensory cues provided by nicotine. Plasma samples to measure MG concentrations were collected before and multiple times after each e-cigarette inhalation. We were particularly interested in (1) whether inhaling e-cigarettes with menthol flavor would lead to changes in MG concentrations, similar to the “menthol boost” observed after smoking cigarettes and (2) whether changes in plasma MG concentrations would predict acute subjective effects from concurrent menthol and nicotine combination. For the second goal, the main outcomes were subjective drug effects, heart rate, and urges to smoke.

## Materials and Methods

### Participants

Out of 57 participants who were enrolled in the parent study (Valentine et al., 2018), 45 (36 men and nine women) provided blood samples and were included in this analysis. Participants were non-treatment-seeking cigarette smokers, ages 18–30, who smoked at least one cigarette per day for the past year. Smoking status was verified with urine cotinine measurements. Both menthol (*n* = 24) and non-menthol (*n* = 21) cigarette smokers were enrolled, and previous experience with e-cigarettes was not required. Participants were medically healthy and were free of major psychiatric disorders including substance use disorders (except tobacco product use), as determined by clinical examination and urine drug screening. Those who used psychotropic medications, were pregnant, were breastfeeding, or had allergies to propylene glycol or menthol were also excluded. Participants provided written informed consent prior to participation and were compensated for their participation. The study protocol was approved by the Institutional Review Boards of the VA Connecticut Healthcare System and Yale University.

### Procedures

This outpatient study, with an adaptation session and three test sessions, utilized a double-blind, placebo-controlled, crossover design (for details, see Valentine et al., 2018). Across the three test sessions, participants were randomized to 0% menthol, 0.5% menthol, or 3.2% menthol conditions (a single menthol condition for each test session), delivered by a standardized inhalation from an e-cigarette just before each IV infusion. In each test session, three IV nicotine infusions (0 mg, 0.25 mg/70 kg, and 0.5 mg/70 kg) were delivered 1 h apart in random order, just after inhalation from e-cigarettes was completed. To minimize carryover menthol effects, the test sessions were performed at least 24 h apart.

### Adaptation Session

The purpose of the adaptation session was to familiarize the participants with the study procedures including the study assessments and the guided e-cigarette inhalation procedure used in the test sessions. A travel-sized menthol-free toothpaste and appointment card with a list of common menthol-containing products were provided as prompts for avoiding exposure to menthol products, other than their usual brand of cigarettes, for 24 h prior to each test session. Participants were also asked to abstain from smoking and eating after midnight prior to all test sessions, but to maintain their usual morning caffeine intake to avoid withdrawal symptoms that might confound interpretation of study measures.

### Test Sessions

Following overnight abstinence from smoking (verified by breath CO <10 ppm), participants attended three test sessions which started between 8 and 9 am. Participants first received a light breakfast, followed by an IV line placement for blood sampling and nicotine infusions.

## Drugs

### Menthol Inhalation

The three different e-liquids were obtained from Pace Engineering Concepts (Delafield, WI, United States). We used Joyetech eGo-C™ e-cigarettes with a single-coil atomizer (2.2 ohm) and a 650 mAh battery operating at 3.7 V (6.2 W). The concentrations of menthol used in this study were based on the ones used in a previous study (Rosbrook and Green, 2016). While the 0.5% concentration is minimally perceptible by smokers, the 3.2% concentration is in the range of commercially marketed mentholated e-cigarette solutions and produces the typical cooling sensation caused by commercial mentholated e-liquids. Each e-liquid contained approximately 25% vegetable glycerin/75% propylene glycol and the same concentration of tobacco flavor. We chose tobacco flavor because of its familiarity to all smokers and its common use as a “control” flavor in e-cigarette studies due to its minimal aversive and appealing qualities (Etter and Bullen, 2011; Kim et al., 2016; Valentine et al., 2018; Leventhal et al., 2019). The concentration of menthol, as well as the absence of nicotine in the three stock e-liquids, was verified in Dr. Peter Jatlow’s laboratory. Participants took six inhalations, 3–4 s each, one inhalation every 15 s, just before each nicotine/saline infusion (Vansickel and Eissenberg, 2013).

### Nicotine Infusion

Nicotine bitartrate was obtained from Interchem Corporation (Paramus, NJ, United States) and was prepared for infusions by the research pharmacy at West Haven VA. Each infusion was delivered over 30 s in a volume of 5 ml. The infusions were given 1 h apart to allow sufficient time for the acute effects of nicotine and menthol to dissipate. The nicotine doses that were chosen for this study have previously been shown to be well-tolerated by both dependent and non-dependent smokers yet also produce robust physiological subjective responses (Sofuoglu et al., 2008; Sofuoglu et al., 2009; Sofuoglu and Mooney, 2009; Sofuoglu et al., 2011; Sofuoglu et al., 2012).

### Outcome Measures

The main outcome measures assessed subjective, cardiovascular, and biomarker domains. Subjective measures were the Drug Effects Questionnaire (DEQ) and the Brief Questionnaire of Smoking Urges (BQSU). The DEQ consists of the following 11 items: cooling effect, dislike the sensation, any sensation (in the mouth, throat, or chest), feel a drug effect, feel high, feel stimulated, feel a head rush, like the drug effect, dislike the drug effect, craving for cigarettes, and would like more of the drug. The DEQ was administered 1, 3, 5, 8, and 10 min following each IV infusion/e-cigarette inhalation. The DEQ assesses acute drug effects by recording response intensity on a 100 mm visual analog scale ranging from “not at all” to “extremely.” The BQSU consists of 10 items rated on a 7-point Likert scale with two factors. While Factor 1 reflects an urge to smoke for stimulation, Factor 2 reflects an urge to smoke to relieve negative mood and withdrawal (Tiffany and Drobes, 1991; Cox et al., 2001). The BQSU was collected at baseline and following each nicotine/e-cigarette administration.

Cardiovascular data consisted of heart rate measurement, which was assessed 5 min before and at 1, 2, 3, 5, 8, 10, and 15 min following each IV nicotine/e-cigarette administration.

Plasma MG concentrations were measured as the biomarker of menthol exposure. Samples were drawn at baseline and at 10, 30, and 60 min following each e-cigarette inhalation. An additional sample was drawn 2 h following the third e-cigarette inhalation. MG was measured by liquid chromatography-tandem mass spectrometry (LC/MS/MS) using MG-d4 (Cambridge Isotope Laboratories, Tewksbury, MA) as an internal standard. The assay was adapted to plasma from that reported for urine MG (Benowitz et al., 2010), with some modification. For sample preparation, we used a protein precipitation/extraction step with methanol rather than simple dilution, and all standards and controls were prepared in MG-free plasma. The lower limit of MG quantitation was 4 ng/ml and between-day reproducibility (CV’s) at 4, 10, and 40 ng/ml concentrations were 17%, 10%, and 8% respectively.

### Data Analyses

Due to the highly skewed nature of the data, nonparametric repeated-measures analysis (Brunner and Langer, 2002) was conducted to evaluate if the maximum change in MG concentrations varied by the participants’ usual cigarette type, (i.e., menthol vs. non-menthol cigarette), or by menthol concentration, or by nicotine dose. The maximum change in MG concentrations was defined as maximum post-dose MG concentrations (10, 30, or 60) minus baseline concentration before inhalation of that dose. Most peak post-dose levels were observed at the 10 min time point. Menthol concentration was a within-subject factor with three levels (3.2%, 0.5%, and 0%), nicotine dose was also a within-subject factor with three levels (0.5 mg/70 kg, 0.25 mg/70 kg, 0 mg/70 kg), and type of cigarettes smoked was a between-subject factor with two levels (menthol vs. non-menthol). All interactions were also tested. We controlled for session and period within-session effects. Peak MG levels were ranked, and mixed models were fit to the ranked data with method-of-moments variance estimators and unstructured variance–covariance matrix within subject. ANOVA-type statistics (ATS) was used to assess statistical significance of the effects in the models.

We used mixed models to evaluate the relationship between changes in MG concentrations and DEQ, heart rate, and BQSU outcomes (Gueorguieva and Krystal, 2004; Brown and Prescott, 2015; Gueorguieva, 2017). Due to the lack of or limited variability in the MG levels at the 0% (no menthol) and 0.5% (lower concentration) menthol conditions, the analyses were restricted to the sessions when 3.2% (higher concentration) menthol was provided. All outcome measures were assessed for normality prior to statistical analysis and transformations were applied to normalize the distributions when necessary.

For the DEQ, peak values for each infusion within each session and participant were calculated. Then the peak values for drug effect, high and stimulated, were averaged to obtain a summary score to represent a composite *stimulatory effects* factor (Morean et al., 2013). Similarly, the peak values for like and want more were averaged to obtain a summary score to represent the *feel good effects* factor. The dislike item by itself was used to represent the *aversive effect*. We also analyzed the *cooling effect* by itself. Peak change in heart rate was calculated as the maximum value following each infusion period minus the baseline value. Factor 1 and Factor 2 of the BQSU were analyzed separately.

All DEQ, BQSU, and heart rate measures were analyzed using mixed-effects models with a between-subject effect of the usual type of cigarettes smoked (menthol vs. non-menthol), within-subject effect of nicotine (saline, low dose (0.25 mg/70 kg), high dose (0.5 mg/70 kg)), within-subject effect of MG peak change (max post-dose–pre-dose baseline, mean centered, as quantitative predictor), and all possible interactions. Session and period were also added to the models. Structured variance–covariance matrices within session were used to model the correlations among repeated measures on the same individual. The best-fitting structure was based on Schwartz-Bayesian information criteria for all outcomes. Slope estimates were used to explain the significant effects of MG peak change in the models. Statistically significant slopes correspond to significant correlations between MG peak change and the outcome.

## Results

### Baseline Variables

With regard to participants’ usual type of cigarettes, non-menthol smokers were more likely to be male and White than menthol-preferring smokers (*p* < 0.05). There were no significant differences in other baseline variables (Table 1).

**TABLE 1:** Baseline demographics and smoking measures by menthol preference.

	Menthol (*N* = 24)		Non-menthol (*N* = 21)	
Characteristic	Number	Percent	Number	Percent
Sex^a^				
Female	8	33.3%	120	4.8
Male	16	66.7%	20	95.2
Race^b^				
White	4	16.7%	19	90.5%
Black	19	79.2%	1	4.8%
Other	1	4.2%	1	4.8
	Mean	Standard deviation	Mean	Standard deviation
Age (years)	24.6	3.4	24.6	3.5
Body mass index	28.1	5.3	25.9	5.6
Age of first smoking	14.6	5.4	15.7	4.0
Cigarettes smoked per day	13.7	7.6	14.5	7.2
Longest estimated abstinence	3.1	4.7	5.0	9.5
FTND^c^ score at baseline	4.3	1.8	4.3	2.2

a Fisher’s exact test indicates significant differences between groups: *p* = 0.02.

b Fisher’s exact test indicates significant differences between groups: *p* < 0.0001.

c FTND: Fagerström Test for Nicotine Dependence.

### Change in Plasma Menthol Glucuronide

**FIGURE 1:**
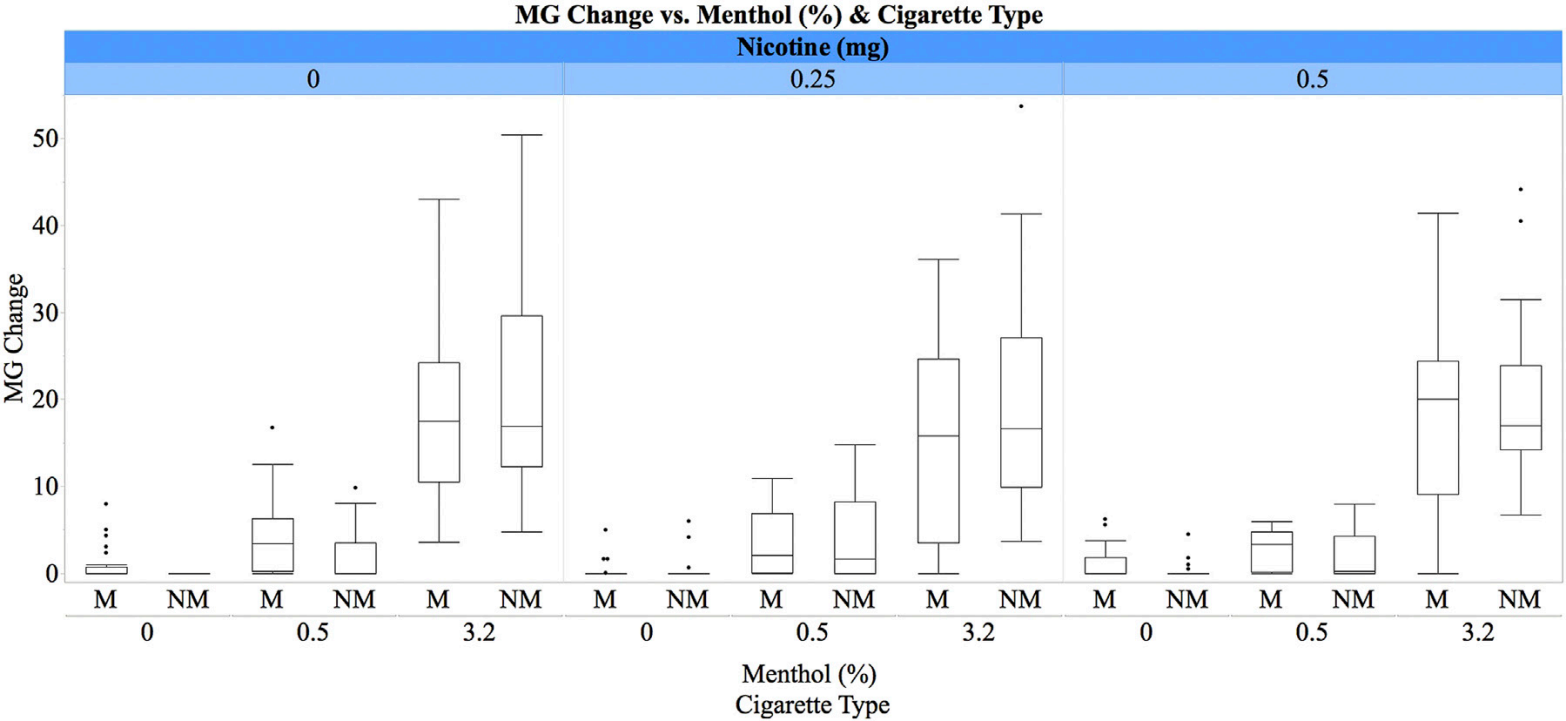
Box plots for plasma MG concentrations (ng/ml) by e-liquid menthol concentrations (0%, 0.5%, and 3.2%), IV nicotine dose (0 mg, 0.25 mg/70 kg, and 0.5 mg/70 kg), and cigarette preference (M = menthol vs. NM = non-menthol). The whiskers represent the minimum and maximum concentrations (excluding outliers), while boxes represent the interquartile ranges (first to third quartiles) with the medians shown as lines inside the boxes. Participants received concurrent inhaled menthol by an e-cigarette and IV nicotine (see text for details).

For the change in plasma MG concentrations following e-cigarette inhalation, there was a significant main effect of menthol concentration [ATS(1.87) = 277.44, *p* < 0.0001], with pairwise comparisons indicating significant concentration-dependent differences [3.2% > 0.5% > 0%]. A significant two-way interaction between usual cigarette type and menthol concentration [ATS(1.87) = 4.98, *p* = 0.008] was noted such that the difference between the changes in MG concentrations for the 3.2% vs. 0.5% menthol was greater for non-menthol than menthol smokers (Figure 1). We also found a significant main effect of session [ATS(2) = 6.53, *p* = 0.002], with lower increases in MG concentrations in the third than the second and first sessions, as well as a significant main effect of period [ATS(2) = 7.00, *p* = 0.0009] with greater increases following the first e-cigarette inhalation than the second and third inhalations. There was also a two-way interaction between nicotine dose and usual cigarette type [ATS(1.86) = 3.98, *p* = 0.02] with significant differences between the 0.5 and 0.25 mg nicotine for smokers of mentholated cigarettes. Menthol smokers had greater changes in plasma MG concentrations under the 0.5 mg condition than under the 0.25 mg nicotine condition (Figure 1).

### Relationship Between Menthol Glucuronide Levels and Drug Effects Questionnaire

**FIGURE 2:**
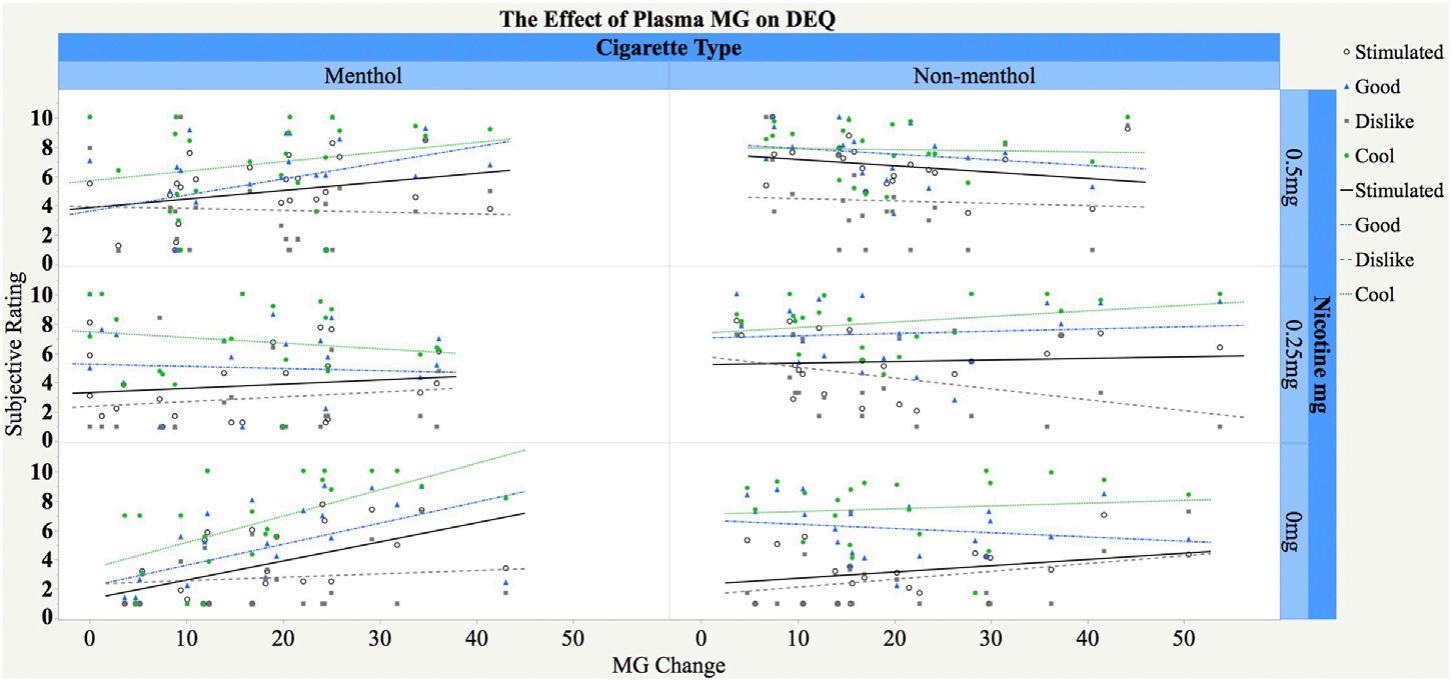
Relationship between change in plasma MG concentrations (ng/ml) and subjective responses to concurrent menthol and nicotine administration in menthol (M) and non-menthol (NM) smokers. The *X*-axis represents increase in MG concentrations (max post-dose–pre-dose baseline), and the *Y*-axis represents post-dose subjective ratings for the three composite scores (*stimulated*, *good*, and *dislike effects*) and *cooling effects*. Participants inhaled menthol by e-cigarette (only 3% included) and received IV nicotine (0 mg, 0.25 mg/70 kg, and 0.5 mg/70 kg) concurrently (see text for details). Fitted regression lines are shown separately by cigarette type and nicotine dose and in different color for the different DEQ measures.

For the DEQ *stimulatory effect*, no significant main effect of MG concentrations, interactions for MG and nicotine, or MG and cigarette preference were found (*p* > 0.05). Consistent with our previous findings (DeVito et al., 2016; Valentine et al., 2018), we observed a main effect of nicotine dose [*F*(2,76) = 41.31, *p* < 0.0001], with dose-dependent [0.5 mg > 0.25 mg > placebo] increases in responses, and nicotine dose-by-cigarette choice interaction [*F*(2,76) = 12.65, *p* < 0.0001], with greater nicotine responses among participants who smoke non-menthol cigarettes (Figure 2).

For the *good effects factor*, no significant main effect of MG, interaction effects of MG and nicotine, or interaction of MG and cigarette preference were found (*p* > 0.05). Consistent with our previous findings (DeVito et al., 2016; Valentine et al., 2018), there were main effects of nicotine [*F*(2,76) = 10.08, *p* = 0.0001] and cigarette preference [*F*(1,41) = 8.98, *p* = 0.005]. Pairwise comparisons indicated a dose-dependent effect of nicotine dose [0.5 mg > 0.25 mg > placebo] as well as greater responses in non-menthol smokers than menthol smokers.

For the *dislike effect*, no main or interaction effect of menthol was found. There was a main effect of nicotine [*F*(2,76) = 7.55, *p* = 0.001], with greater “dislike” under higher doses [0.5 mg > 0.25 mg > placebo].

For the *cooling effect*, there was a significant three-way interaction between changes in MG concentrations, usual cigarette type, and nicotine dose [*F*(2,76) = 4.53, *p* = 0.01]. Post-hoc tests showed that the slope for the relationship between MG change and *cooling effect* under 0% nicotine for menthol smokers was significantly greater than 0 such that a larger MG change was associated with significantly greater cooling ratings [slope = 0.11, SE = 0.04, *t*(76) = 2.99, *p* = 0.004]. The rest of the slopes were not significantly different from 0.

### Relationship Between Menthol Glucuronide Levels and Smoking Urges

For the BQSU Factor 1 or Factor 2, no main or interactive effects of MG were observed (*p* > 0.05). For Factor 1, there was a main effect of cigarette preference [*F*(1,41) = 5.85, *p* = 0.02], with lower scores in menthol smokers than non-mental smokers and a significant main effect of nicotine [*F*(2,78) = 3.12, *p* = 0.05], consistent with our previous studies (DeVito et al., 2016; Valentine et al., 2018).

### Relationship Between Menthol Glucuronide Levels and Heart Rate

For changes in heart rate, no main or interactive effects of MG were observed (*p* > 0.05). Consistent with our previous work, there was a main effect of nicotine [*F*(2,73) = 8.6, *p* < 0.001], and pairwise comparisons showed a dose-dependent increase in heart rate [0.5 mg > 0.25 mg > saline].

## Discussion

The purpose of this exploratory study was to examine if changes in plasma MG concentrations were predictive of acute subjective and cardiovascular effects of intravenous nicotine and inhaled menthol. The study had several notable findings. First, following e-cigarette inhalation, changes in plasma MG concentrations or the “menthol boost” increased proportionally to the menthol concentration in the e-liquids. Second, while changes in plasma MG concentrations were not predictive of DEQ items that are reflective of acute effects from nicotine (e.g., *stimulated* and *good drug effects*), they were predictive of *cooling effect*, a typical subjective effect of menthol, but only in menthol smokers in the absence of concurrent active nicotine infusion (i.e., in the placebo condition). Third, changes in plasma MG concentrations were not predictive of heart rate increases following menthol and nicotine administration. Although these findings do not support the predictive role of plasma MG concentrations for subjective and heart rate responses to concurrent exposure to nicotine and menthol, they demonstrate the utility of plasma MG as a biomarker for acute menthol exposure via e-cigarette inhalation. To our knowledge, this is the first study that examined if plasma MG concentrations were predictive of the acute subjective and physiological effects of menthol and nicotine.

In previous studies, urine MG concentrations were shown to be a biomarker for mentholated cigarette smoking status (Benowitz et al., 2010). Furthermore, following menthol cigarette smoking, increases in plasma MG concentrations have also been demonstrated (Hsu et al., 2017). In a prior study, we showed that the AUC values of plasma MG concentrations increase proportionally to the concentration of menthol in the e-liquid (Jatlow et al., 2018). In this study, we demonstrate changes in plasma MG concentrations following inhalation of e-cigarettes with different menthol concentrations. Menthol is rapidly metabolized to MG and other oxidative metabolites, and menthol itself is not detected in plasma following its oral administration (Gelal et al., 1999). MG accounts for about half of the menthol concentration. The concentration-dependent increase in MG levels supports the utility of plasma MG as a biomarker for menthol inhalation from e-cigarettes. The use of MG can especially be helpful in behavioral studies considering the large interindividual variation of smoking patterns of tobacco products like cigarettes and e-cigarettes. Use of MG as a biomarker of acute menthol exposure will have utility in better understanding the acute effects of menthol across a broad range of outcomes.

The lack of predictive effects of plasma MG concentrations for the acute subjective responses to concurrent nicotine and menthol administration were consistent with the main study where inhaled menthol at different concentrations did not change the subjective effects of the IV nicotine (Valentine et al., 2018). One exception was the rating of *cooling effect*, which was observed only in menthol smokers in the absence of active nicotine, suggesting that concurrent nicotine might have suppressed the *cooling effect*. The doses of nicotine that were used in this study (0.25 mg/70 kg and 0.5 mg/70 kg) are less than the amount of nicotine delivered by smoking a cigarette, which ranges from 0.5 to 2 mg (about 0.05–0.2 mg/puff) (Carmines and Gillman, 2019). However, as we reported recently, these doses are significantly greater than the threshold doses of IV nicotine for its subjective and reinforcing effects, 0.05 and 0.2 mg, respectively (MacLean et al., 2021). Whether menthol’s effects on the subjective responses to nicotine may be different in lower dose ranges of nicotine remains to be determined in future studies.

Plasma MG concentrations did not predict heart rate increases following concurrent menthol and nicotine administration. These findings are consistent with a previous study which reported that the acute heart rate increases following smoking of mentholated cigarettes were not different from those following smoking of non-mentholated cigarettes (Pickworth et al., 2002). Another noteworthy finding was that the change in plasma MG concentrations, “menthol boost,” was greater for non-menthol than menthol smokers. It is possible that these differences are due to acceleration of menthol clearance by chronic menthol exposure (Ahijevych and Garrett, 2004). In addition, menthol smokers, but not non-menthol smokers, had greater MG concentrations when menthol inhalation was combined with the 0.5 mg nicotine condition than with the 0.25 mg nicotine condition. It is unclear if these results are due to greater menthol inhalation under the 0.5 mg nicotine condition or pharmacokinetic interaction between nicotine and menthol. Because menthol inhalation (via e-cigarette) preceded nicotine infusions, it is unlikely that nicotine infusion could have impacted the e-cigarette inhalation behavior. We are also not aware of any pharmacokinetic interaction between acute nicotine and menthol that can explain these findings. These possibilities remain to be examined in future studies.

The strengths of our study included the use of multiple menthol e-liquid concentrations using a directed e-cigarette inhalation, as well as the ability to ensure precise dosing and timing of nicotine delivery by use of the intravenous nicotine delivery paradigm. Despite the benefits of the IV nicotine delivery paradigm (e.g., Jensen et al., 2016), this approach does limit the ability to account for the oral and respiratory effects of nicotine (e.g., bitter taste and “throat hit”). Menthol is thought to increase the appeal of nicotine-containing tobacco products, at least in part, by masking or counteracting these aversive or harsh effects of nicotine in the mouth and throat (DeVito et al., 2020). The lack of significant correlations between “menthol boost” and subjective effects of nicotine delivery could, in part, be accounted for by this limitation in the study design. Future studies could assess the relationship between “menthol boost” (as confirmed by increased plasma MG concentrations) and subjective response to vaped or smoked nicotine. Another limitation was the small number of women participating in the study, which precluded examination of sex differences. In addition, unbalanced race distribution across menthol and non-menthol smokers did not allow examination of the independent contribution of race on study outcomes. Another limitation was the inclusion of only two doses of nicotine that were higher than the threshold doses for nicotine reinforcement. Finally, the study recruited daily smokers and did not differentiate between levels of nicotine dependence. These limitations could be addressed in future studies. For example, randomization could be balanced on gender and race, which would allow us to control for the potential confounding effects of these factors and also to estimate moderator effects. Additional menthol and nicotine doses could also be included.

Our study has important implications for tobacco regulatory science. These findings support the validity of repeated measures of plasma MG concentrations and indicate that changes in MG concentration can capture sensitivity to dose delivery and relate to the subjective experience of menthol levels (i.e., “cooling” effects). Menthol is the only characterizing flavor permitted in cigarettes and is also commonly added to e-liquids. The impact of menthol on the development and maintenance of tobacco product use continues to be an important public health problem. However, there are important gaps in our knowledge on menthol’s actions in humans, especially in relation to its role in enhancing nicotine’s rewarding and reinforcing effects. Such studies may benefit from plasma MG concentrations that can help to address individual differences in the amount of menthol exposure and as a result a more accurate understanding of menthol’s biological and behavioral effects in humans. Given individual differences in vaping or smoking topography and the vast array of tobacco products (including a wide range of e-cigarette devices) available on the market, which delivers aerosol/smoke at different rates, it is difficult to precisely control the amount of aerosol/smoke constituents delivered to a subject in human laboratory studies. The current findings suggest plasma MG concentration measurements could serve as a useful measure of the amount of menthol actually delivered to the user in research paradigms (or in naturalistic use settings).

In summary, this study demonstrates that following inhalation of e-cigarettes, changes in plasma MG concentrations or “menthol boost” increased proportionally to the menthol levels in e-liquids. Plasma MG concentrations were not predictive of heart rate or acute subjective effects of nicotine, except *cooling effect*, a typical subjective effect of menthol. These findings demonstrate the utility of plasma MG as a biomarker for acute menthol exposure via e-cigarette inhalation.

## Data Availability

The raw data supporting the conclusion of this article will be made available by the authors, without undue reservation.
